# Cranial Cruciate Ligament Desmotomies in Sheep Resulting in Peroneus Tertius Injury

**DOI:** 10.1155/2021/2628791

**Published:** 2021-07-21

**Authors:** Peter J. Welsh, Crystal G. Collier, Holly M. Clement, Michael N. Vakula, Jeffrey B. Mason

**Affiliations:** ^1^School of Veterinary Medicine, Utah State University, 4815 Old Main Hill, Logan, UT 84322-4815, USA; ^2^Department of Animal, Dairy, and Veterinary Sciences, Utah State University, 4815 Old Main Hill, Logan, UT 84322-4815, USA; ^3^Department of Kiniseology, Utah State University, 7000 Old Main Hill, Logan, UT 84322-7000, USA

## Abstract

Surgical destabilization of the stifle joint via cranial cruciate ligament desmotomy (CCLD) is a routine procedure for the study of osteoarthritis (OA). Traditionally performed in rats, rabbits, cats, and dogs, CCLD in sheep provides an opportunity to study the pathology and treatment of joint instability in a species whose stifle better represents the equivalent human femorotibial joint. The surgical approaches for CCLD in sheep are variable and can result in inconsistent outcomes. Eight sheep underwent CCLD for use in a gene therapy study. We report this case in which six of the eight sheep were clinically diagnosed by pathognomonic signs and later confirmed by postmortem dissection, with injury of the peroneus tertius (PT) muscle. The PT plays a crucial role in the normal gait of large animals, including sheep. Injury to the PT results in failure of the reciprocal apparatus of the hind limb in which the hock can be extended during stifle flexion creating a varied gait and an indiscriminate increase in instability of the stifle and hock joints. Restricted movement postoperatively may provide decreased variability in surgical outcomes. Alternatively, increased stifle instability via CCLD coupled with PT transection or PT transection alone could potentially provide a superior model of stifle instability and OA development in sheep.

## 1. Introduction

In small ruminants, the procedure of surgically transecting the cranial cruciate ligament (CCL), known as a cranial cruciate ligament desmotomy (CCLD) or anterior cruciate ligament transection, has been shown to induce osteoarthritis (OA) [1–4]. Sheep are often preferred for the CCLD model due to their ease of care, but also their articular anatomy, which closely resembles that of the human knee [5]. Unique in structure to large animals, the peroneus tertius (PT) muscle shares a proximal fused tendinous origin with the extensor digitorum longus and extensor digitorum medialis at the extensor fossa of the femur [6]. It is likely that the fused tendon of the PT, extensor digitorum longus, and extensor digitorum medialis contributes to stifle stability by preventing cranial subluxation of the tibia; however, this phenomenon and the biomechanics unique to these structures require further investigation and statistical validation [4].

Complications when accessing the CCL intraoperatively via arthrotomy and the inability to fully limit activity during recovery are two variables that may lead to unanticipated intra- or postoperative injury to supporting structures of the stifle joint, such as the PT muscle/tendon. The CCL can be accessed via a medial or lateral parapatellar arthrotomy [6]. However, despite a moderate learning curve, arthroscopy for CCL access has provided easier access to the joint, enhanced tissue visibility, and decreased tissue damage [7, 8]. Despite intraoperative trauma following CCLD, postoperatively sheep are often immediately housed in large pens or open pasture and are rarely limited in their activity [4, 7, 8]. Unrestricted movement leaves potential for injury and other postoperative complications.

## 2. Case Presentation

Eight Suffolk cross sheep were selected randomly from a group of two to three-year-old intact females with normal orthopedic conformation to undergo CCLD via a modified Pond-Nuki technique as part of a study for novel gene therapy. Medial or lateral parapatellar arthrotomy was used to access and transect the CCL per the surgeon's preference as previously reported [1–4]. All CCLDs were performed on the left stifle.

**Figure 1:**
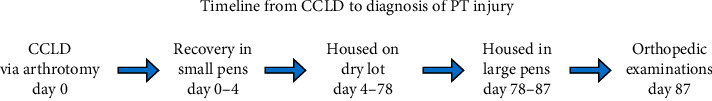
Timeline from CCLD to the diagnosis of PT injury. After postoperative recovery for five days, sheep were given unrestricted mobility in a dry lot for 74 days and received comprehensive orthopedic exams at day 87 after CCLD.

Sheep were recovered in a small group stall (8 ft × 10 ft) bedded in straw for five days before being moved to a large dry lot with no mobility restrictions (Figure 1). Recovery was unremarkable in all sheep.

**Figure 2:**
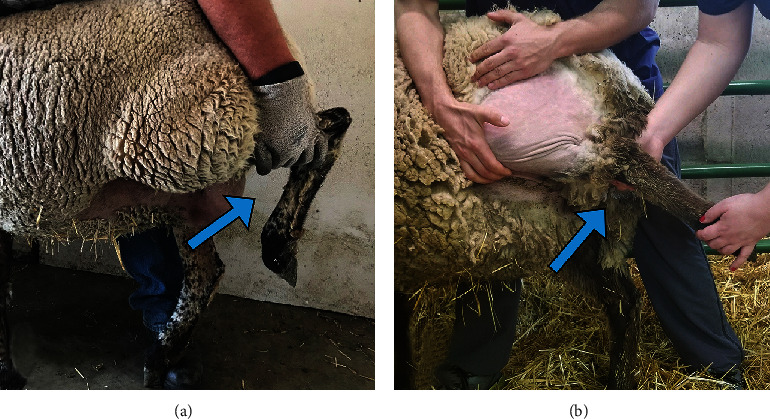
Normal antemortem stifle flexion compared to that of a sheep with an injured peroneus tertius. Damage to the PT in six out of eight sheep that underwent CCLD for use in a gene therapy study was suspected based on clinical signs (b) when compared to healthy unaffected sheep (a).

During intraoperative and/or postoperative examination, six of eight CCLD subjects were found to have an abnormal extension of the hock joint during simultaneous flexion of the stifle joint in the affected limb (AL) (Figure 2(b)) compared to an unaffected limb (Figure 2(a)). These findings suggest intra- and/or postoperative injury to the PT. All sheep in the study were subjected to oblique angle forced exercise, serial radiographic examinations, and were humanely euthanized upon the completion of the study for tissue analysis.

Antemortem radiographs were performed to monitor for radiographic evidence of OA, and postmortem gross analysis was performed to diagnose and characterize the injury and to discern how it may have occurred. Radiographic findings showed no correlation to the clinical history of PT injury. Postmortem pelvic limb dissection revealed focal injury to the proximal tendon of the PT in all affected sheep.

### 2.1. Materials and Methods

Materials and methods described were derived from the gene therapy research protocol from which this case report stemmed.

#### 2.1.1. Sheep

Mature Suffolk-cross ewes, two and three-year-old, with body condition scores between 3 and 4 out of 5 (on a scale of 1 to 5 with 1 being emaciated and 5 being extremely obese) and no observable musculoskeletal disease, were used in a novel gene therapy study. Sheep were fed twice daily with water ad libitum throughout the study. Sheep were initially housed in small group pens for the first five days postoperatively. After the immediate postoperative period, sheep were then housed in a dry lot for approximately 74 days before being group housed in large (16 ft × 32 ft) indoor pens with unrestricted mobility for the remainder of the study. Housing, surgical procedures, treadmill exercise, radiographic imaging, in vivo sample collection, and monitoring were all conducted at a United States Department of Agriculture Agricultural Research Service (USDA ARS) facility. Macroscopic soft tissue analysis was conducted at an associated veterinary diagnostic laboratory (blinded).

#### 2.1.2. Ethical Treatment of Animals

Animal care and use protocols were developed under the Animal Welfare Act and Animal Welfare Regulations guidelines and approved by the Utah State University Institutional Animal Care and Use Committee (IACUC-10046) and were carried out in accordance with all applicable institutional, local, and national guidelines.

#### 2.1.3. Surgical Procedure

Surgical destabilization in the ewes was accomplished using a CCLD as previously described [1–4]. All surgical procedures were conducted at the USDA ARS facility under the approval of the IACUC by the same surgeon in all cases. The sheep were sedated for anesthesia using xylazine (AnaSed, 0.07 mg/kg intravenous, Akorn, Lake Forest, IL) and acepromazine (acepromazine, 0.13 mg/kg intravenous, VetOne, MWI Animal Health, Boise, ID). Intravenous flunixin meglumine (Prevail, 2.2 mg/kg intravenous, VetOne, MWI Animal Health, Boise, ID) was given for analgesia, and cefazolin (Anacef, 1 g/sheep intravenous, West-Ward Pharmaceuticals Corp, West Eatontown, NJ) was given as a preoperative antibiotic. For postoperative analgesia, all animals received meloxicam (meloxicam 1 mg/kg orally, Zydus Pharmaceuticals, Pennington, NJ) immediately prior to induction, again 24 hours later, then as needed on a case by case basis. Ten minutes following administration of sedation, all sheep were given ketamine (Zetamine, 14 mg/kg intravenous, VetOne, MWI Animal Health, Boise, ID) for induction followed by orotracheal intubation and gas anesthetic for maintenance using 1-2% isoflurane in 100% oxygen. Using standard sterile surgical techniques, an arthrotomy medial or lateral to the middle patellar ligament was performed exclusively on the left stifle of all sheep according to the associated gene therapy study design. All CCLDs were completed by the same surgeon, and all procedures were done within a two-day time period to limit intraoperative variability. The surgeon used a medial or lateral approach on a case by case basis. Following arthrotomy, the cranial tibial attachment of the medial meniscus and cranial cruciate ligament was identified. Debakey forceps were passed behind the CCL for isolation, and transection was performed with a #11 scalpel blade. Complete CCL transection was confirmed by a positive cranial drawer sign. Prior to closure, the joint was lavaged with sterile 0.9% saline. The joint capsule and associated connective tissue were closed using simple interrupted 1 absorbable braided suture (Vicryl Ethicon, Cincinnati, OH). The subcutaneous tissue was closed using 2-0 absorbable braided suture (Vicryl Ethicon, Cincinnati, OH) in a continuous pattern, and the final layer was closed using surgical staples (Weck Visistat 35R, Teleflex Medical, Research Triangle Park, NC). Aluminum bandage spray (AluSpray, Neogen Animal Safety, Lexington, KY) was applied to the surgical site. Recovery from anesthesia was uneventful for all sheep. Upon recovery, sheep were housed in groups of two to three in 8 ft.×10 ft. indoor pens bedded in straw for five days then moved to a large dry lot with free mobility (Figure 1).

#### 2.1.4. Physical Examination

Sheep were monitored at least twice daily for signs of postoperative complications such as pain, fever, and/or infection at the surgery site. Skin staples were removed at ~14 days following surgery. Complete physical exams for analysis of surgical recovery, cardiovascular and respiratory health, and musculoskeletal soundness were performed 87 days following CCLD. The common tendon of the PT, extensor digitorum longus, and extensor digitorum medialis originates from the extensor fossa on the craniolateral aspect of the proximal femoral condyle and inserts on the extensor process of the distal phalanx [6]. This anatomy results in coordinated movement in unison between the hock and stifle joints when the PT is intact (Figure 2(a)).

#### 2.1.5. Exercise

**Figure 3:**
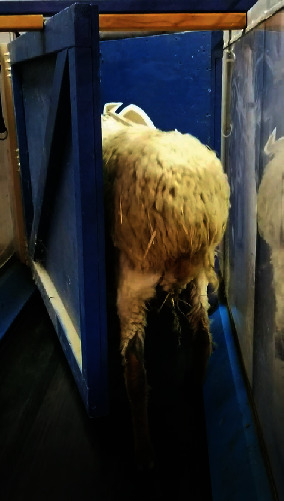
After CCLD, sheep were subjected to oblique angle forced exercise by treadmill for the exacerbation of osteoarthritis as part of a novel gene therapy study.

After surgical destabilization and a period of 98 days, sheep were subjected to oblique angled (35°) forced exercise on a treadmill (Horse Gym 200 Walk S3, Horse Gym USA®, Wellington, FL) at 80 M/min for 32 min (Figure 3), every other day prior to morning feeding for increased mechanical stress on the stifle joint as previously reported [9].

#### 2.1.6. Gait Analysis

Between days 112 and 119 of forced exercise, two-dimensional kinematics was recorded using a video camera (iPhone 7, Apple Inc., Cupertino, CA) recording at 120 Hz. The sheep walked on the treadmill in a straight path at 80 M/min for a minimum of 1 minute in order to capture 20 acceptable gait cycles. The stance time of each limb was extracted using video analysis software (Kinovea Inc.), and the average stance times were used for analysis.

#### 2.1.7. Radiographic Imaging

Restrained with halter and lead rope, all sheep were fully weight-bearing in a standing position for each radiographic study. Four total views of both the left and right stifle at each imaging time point were taken and consisted of a (1) caudocranial, (2) lateromedial, (3) caudo 60° lateral-craniomedial oblique, and (4) flexed skyline view. Radiographs were taken at days 77, 119, 161, and 203 post-CCLD. From each image, the evaluation of the extensor fossa for pathology related to the proximal PT attachment was determined.

#### 2.1.8. Macroscopic Soft Tissue Analysis

**Figure 4:**
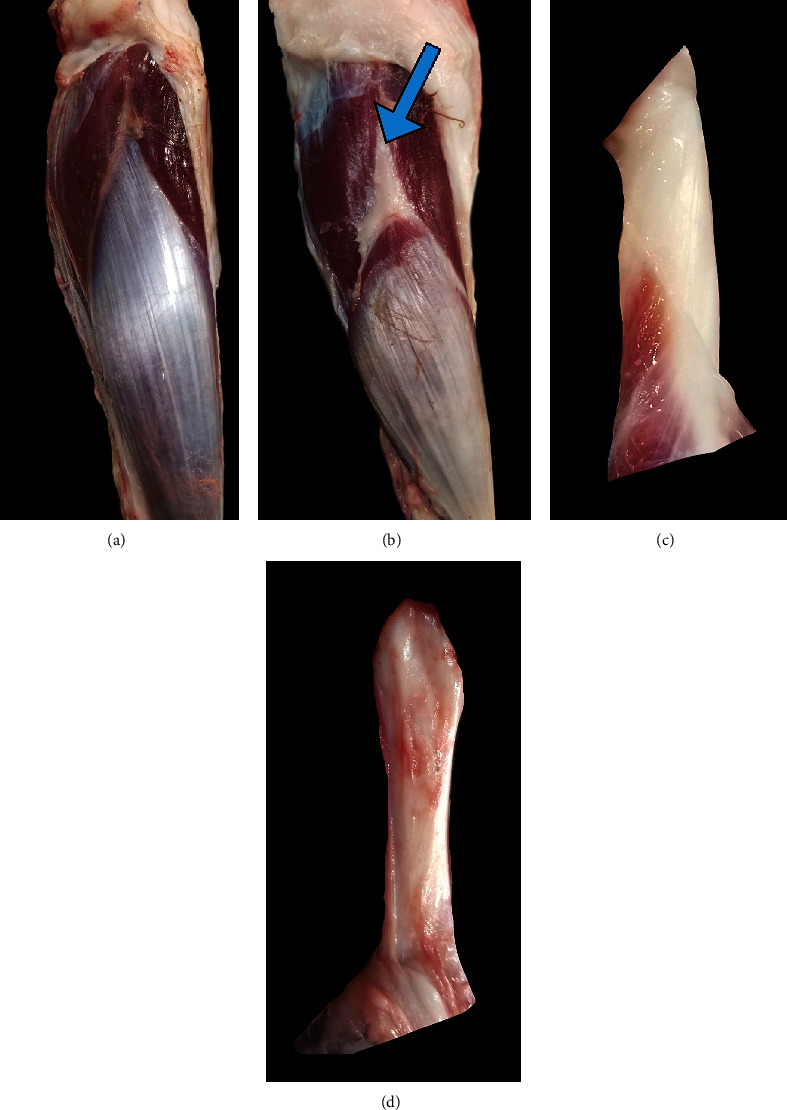
Gross changes in the peroneus tertius. Damage to the PT in six out of eight sheep that underwent CCLD was confirmed via postmortem dissection when compared to healthy unaffected sheep ((a) intact and (b) disrupted). Gross structural changes can also be seen when comparing an intact proximal PT tendon (c) to a damaged proximal PT tendon (d). This may lead to variable joint stability between test subjects.

The sheep were euthanized with an intravenous overdose of pentobarbital/phenytoin (Euthanasia Solution, 100 mg/kg, VetOne, MWI Animal Health, Boise, ID). Clinically normal sheep PT muscle and tendons (Figures 4(a) and 4(c)) were grossly compared to affected sheep (Figures 4(b) and 4(d)) to determine the location and extent of the tissue damage. Proximal and distal PT tendons were transected where they entered the stifle joint capsule and hock, respectively, and percent change in muscle belly length was measured between affected and intact PT muscles.

#### 2.1.9. Data Analysis

The small sample size in our report does not provide enough power to perform inferential statistics on the collected data. Instead, descriptive statistics depicted as percentages of averages were used to illustrate the observed changes in our subjects for gait analysis and macroscopic soft tissue analysis.

### 2.2. Results

#### 2.2.1. Physical Examination

Clinical signs of PT injury were first observed in a single sheep intraoperatively. Extension was possible in the hock joint while the stifle was simultaneously held in flexion (Figure 2(b)). At the time of complete physical examination, 87 days after CCLD, those same clinical signs were observed in five more of the eight sheep that experienced CCLD. The ability to extend the hock while the stifle is flexed is a pathognomonic physical examination finding for disruption of the PT [10, 11].

#### 2.2.2. Exercise

All sheep, including those with PT injury, completed all required exercises with no complications.

#### 2.2.3. Gait Analysis

Stance time was 3.7% longer in the surgically destabilized left pelvic limb in comparison to the contralateral unaffected right pelvic on average regardless of PT injury. However, the average stance time of the AL was 7.2% shorter in comparison to sheep without PT injury.

#### 2.2.4. Radiographic Images

No radiographic evidence for PT rupture was observed in affected sheep at the extensor fossa as has been reported in horses with similar injuries [10].

#### 2.2.5. Macroscopic Soft Tissue Analysis

Soft tissue evaluation during pelvic limb dissection revealed that the PT tendons were approximately 40 to 50% smaller in diameter and had a tan color in the AL (Figures 4(c) and 4(d)). While the muscle belly and tendon remained intact, the proximal PT tendon was stretched or displaced distally approximately 15 to 20% of the original length such that the PT muscle belly of the AL was only 80 to 85% the length of an unaffected PT (Figures 4(a) and 4(b)).

## 3. Discussion

Physical exam findings were conclusive that six out of eight sheep that underwent unilateral left CCLDs also experienced damage to the left PT. There was no correlation between surgical order or technique and incidence of PT injury upon review of surgery reports. The clinical signs of hock extension during simultaneous stifle flexion were initially seen in one sheep intraoperatively while the remaining five sheep were diagnosed 87 days postoperatively and prior to oblique angle forced exercise. Soft tissue analysis upon pelvic limb dissection revealed injury to the proximal PT tendon.

Oblique angled forced exercise was designed to exacerbate joint stress [9]; however, the specific biomechanics regarding how this increases stress on the stifle has not been described or quantified. Moreover, no sheep were run in an unangled/linear path as a control in the associated gene therapy study. It is likely that the oblique angle design accounts for the increased stance time of the surgical left leg compared to the unaffected right leg (Figure 3). However, the decrease in stance time observed in the AL when compared to the same limb in sheep that underwent CCLD without PT injury is suggestive of off-loading of the AL. Though off-loading has not been quantified or readily described in sheep, it is a common sign of musculoskeletal pain or instability. Although a visibly altered gait was observed during oblique angled forced exercise, pelvic limb stance time between sheep with and without PT injury was only marginally different. The small but measurable difference in stance time suggests PT injury results in a compensatory gait strategy that reduces stance time on the AL when compared to the unaffected limb.

To the authors' knowledge, PT injuries, traumatic or iatrogenic, in sheep have not been reported. However, in a study of PT tears in 27 horses (21 related to trauma and 6 from laceration), rupture occurred in the midbody of the tendon in 11, at the site of insertion in 11, and at the origin in 2 horses [10]. Horses with rupture at the PT origin also experienced concurrent rupture of the extensor digitorum longus tendon and avulsion fracture at the lateral femoral condyle. The low incidence of rupture at the origin of the PT in horses and the lack of observed fracture avulsion at the lateral femoral condyle in the AL may suggest that the PT injuries in this case are more likely related to complications during surgery rather than spontaneous traumatic rupture. However, considering the retrospective nature of injury diagnosis, the exact etiology for PT injury in this case study is left to speculation.

Normal treatment protocol for PT rupture is extended stall rest until the onset of fibrosis. In studies where OA development is intentionally provoked, stall rest may not be appropriate. Unanticipated damage to supporting structures of the stifle, like the PT, may result in variable joint stability and biomechanics of test subjects possibly leading to inconsistent development of OA severity.

Though we could not identify the exact cause of the PT injury, the size and location of the proximal PT tendon suggest three points that should be considered for future ovine CCLD studies:

1. During CCLD, special care should be given to prevent unintended damage to the proximal PT tendon and other supporting structures when accessing the CCL. Arthroscopic visualization and transection of the CCL may limit tissue damage and reduce risk of damage to these supporting stabilizing structures
2. The patient should be evaluated by assessing the range of motion through all pelvic limb joints for failure of the reciprocal apparatus intraoperatively and at frequent time points postoperatively to better characterize when failure/PT injury becomes evident
3. Postoperative care for subjects that undergo CCLD should include extended stall rest to minimize the potential for acute trauma of the PT during recovery

Alternatively, as suggested by a previous study, increased stifle instability via CCLD coupled with PT transection or PT transection alone could potentially provide a superior model of stifle instability and OA development in sheep [4].

## Data Availability

The datasets used and/or analyzed during the current study are available from the corresponding author on reasonable request.
